# Terpenoid Hydrazones as Biomembrane Penetration Enhancers: FT-IR Spectroscopy and Fluorescence Probe Studies

**DOI:** 10.3390/molecules27010206

**Published:** 2021-12-29

**Authors:** Mariia Nesterkina, Serhii Smola, Nataliya Rusakova, Iryna Kravchenko

**Affiliations:** 1 Department of Organic and Pharmaceutical Technologies, Odessa National Polytechnic University, 65044 Odessa, Ukraine; kravchenko.pharm@gmail.com; 2 A.V. Bogatsky Physico-Chemical Institute, National Academy of Sciences of Ukraine, 65080 Odessa, Ukraine; sssmola@gmail.com (S.S.); natavrusakova@gmail.com (N.R.)

**Keywords:** terpenes, hydrazones, penetration enhancers, liposomes, lipids, stratum corneum, laser diffraction, fluorescence probe, pyrene, FT-IR spectroscopy

## Abstract

Hydrazones based on mono- and bicyclic terpenoids (verbenone, menthone and carvone) have been investigated in vitro as potential biomembrane penetration enhancers. In this regard, liposomes composed of lecithin or cardiolipin as phospholipid phase components with incorporated fluorescence probes have been prepared using the thin-film ultrasonic dispersion method. The mean particle size of the obtained liposomes, established using laser diffraction, was found to be 583 ± 0.95 nm, allowing us to categorize them as multilamellar vesicles (MLVs) according to their morphology. Pursuant to fluorescence analysis, we may assume a reduction in microviscosity and, consequently, a decrease in the packing density of lecithin and cardiolipin lipids to be the major mechanism of action for terpenoid hydrazones **1**–**15**. In order to determine the molecular organization of the lipid matrix, lipids were isolated from rat strata cornea (SCs) and their interaction with tested compounds was studied by means of Fourier transform infrared spectroscopy. FT-IR examination suggested that these hydrazones fluidized the SC lipids via the disruption of the hydrogen-bonded network formed by polar groups of SC constituents. The relationship between the structure of terpenoid hydrazones and their ability to enhance biomembrane penetration is discussed.

## 1. Introduction

The development of novel drug molecules involves not only the interaction of the compounds with their potential pharmacological targets but also a mechanism of drug delivery that can overcome biological barriers such as the skin, blood–brain barrier and cell and nuclear membranes. Many pharmacologically active compounds exhibit low activity when administered orally due to their reduced bioavailability, largely caused by poor membrane permeability [1]. Transdermal delivery or topical applications are limited by the stratum corneum barrier, which is the skin’s outermost layer, comprising cells compressed into a matrix of intercellular lipids [1,2]. In order to improve the membrane permeability of hydrophilic, low-molecular-weight compounds, the chemical modification of their structure can be applied. The prodrug strategy serves as an example of this approach, consisting of drug molecule derivatization, leading to the facilitation of the permeability of compounds and their subsequent enzymatic cleavage [3]. Such a method of increasing lipophilicity and prolonging action has been successfully employed for morphine, naltrexone, ketorolac, bupropion, theophylline and haloperidol [4,5,6,7,8,9]. More impactful, however, is a multidrug idea aimed at the transporting of hydrophilic drugs through membrane barriers through their conjugation with bioactive compounds possessing penetration enhancer (PE) properties. Among various PEs, terpenes and their oxygen-containing derivatives have received much attention due to their high efficiency, safety, low skin irritation and their ability to improve the permeability of both lipophilic and hydrophilic molecules [10]. Notably, terpenes demonstrate their own biological and pharmacological activity: antidepressant, antidiabetic, anticancer, anticonvulsant, antiviral, analgesic, anti-inflammatory and antioxidant effects [11,12,13,14]. Terpenoid scaffolds have been used to improve the membrane permeability of hydrophilic neurotransmitter amino acids (GABA and glycine); as a consequence, esters with multi-target activities and the ability to disrupt lipid packing have been synthesized [15,16]. 

The concept of multidrugs (or polypharmacology) was successfully implemented by our research group when searching for novel compounds capable of simultaneously affecting the central and peripheral nervous systems. In this respect, oxygen-containing terpenes, as agonists of transient receptor potential (TRP) channels and allosteric modulators of GABA_A_ receptors [17,18], were conjugated with *para*-substituted phenoxyacetic acid hydrazides that also manifest anticonvulsant and nociceptive potentialities [19,20]. Therefore, hydrazones based on carvone, menthone and verbenone were obtained and tested in vivo as potential antiseizure and analgesic agents with prolonged action due to the enzymatically degraded azomethine –NH–N=C– group in their structure [21,22,23]. Bearing in mind the high pharmacological effect of the aforementioned terpenoid derivatives with oral administration and topical applications, studies of their impact on lipid molecular organization are feasible. Some main mechanisms of permeation enhancement involve the disruption of lipid bilayer packing, transient opening of tight junctions, complexation/carrier/ion pairing and disruption of the cellular protein structure [1]. In order to elucidate the mechanism of interaction between enhancers and artificial membranes or extracted lipids, the fluorescence probe method and Fourier transform infrared spectroscopy (FT-IR) have been extensively adopted [24,25].

Given the above, current paper is devoted to the investigation of the use of terpenoid hydrazones as biomembrane enhancers on models of artificial phospholipid membranes and lipids isolated from the stratum corneum (SC), exploiting fluorescence studies and Fourier transform infrared spectroscopy.

## 2. Results and Discussion

### 2.1. Pyrene Fluorescence Studies

**Figure 1:**
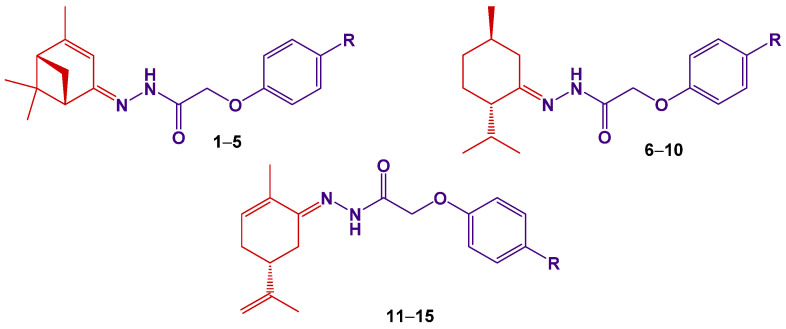
Structures of hydrazones based on (‒)–verbenone (**1**–**5**), (‒)–menthone (**6**–**10**) and (‒)–carvone (**11**–**15**). R = H (**1**, **6**, **11**); R = Br (**2**, **7**, **12**); R = Cl (**3**, **8**, **13**); R = C(CH_3_)_3_ (**4**, **9**, **14**); R = O–C_6_H_5_ (**5**, **10**, **15**).

According to the concept of polypharmacology, drug molecules may simultaneously interact with multiple targets, thereby interfering with multiple disease pathways [26]. This, in turn, contributes to avoiding the polypharmacy phenomenon that is defined as the use of multiple medications by patients with multimorbidity [27]. Developing the idea of polypharmacology (or multidrugs), we previously observed compounds contemporaneously affecting both the central and peripheral nervous systems. To that end, hydrazones based on verbenone (**1**–**5**), menthone (**6**–**10**) and carvone (**11**–**15**) containing the residues of para-substituted phenoxyacetic acid were synthesized (Figure 1).

The aforementioned terpenoid derivatives were found to exhibit anticonvulsant activity on pentylenetetrazole and maximal electroshock seizure test results, along with analgesic potency on chemically and thermally-induced pain models [21,22,23]. Expanding the concept of polypharmacology, we attempted to design molecules capable of modulating different types of receptors and enhancing self-permeability across biological barriers after their oral administration or transdermal/topical delivery. Mono- and bicyclic oxygen-containing terpenes were selected as scaffolds for the synthesis of novel derivatives due to their high enhancement effect, safety and low skin irritation. In order to investigate the membrane permeability in vitro, liposomes are widely utilized as simple cell models because of their similarity to cellular membranes [28]. In the current study, liposomes composed of lecithin or cardiolipin as phospholipid phase components with incorporated fluorescence probes and terpenoid hydrazones **1**–**15** were prepared using the thin-film ultrasonic dispersion method.

**Figure 2:**
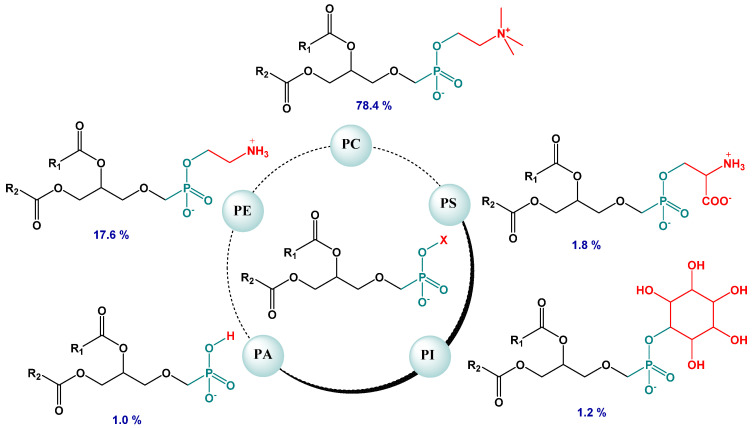
Phospholipid composition of egg yolk lecithin (%): PC—phosphatidylcholine, PE—phosphatidylethanolamine, PI—phosphatidylinositol, PA—phosphatidic acid, PS—phosphatidylserine.

In the present study, egg yolk lecithin, representing a multi-component system, was applied. Concerning its composition, the following constituents should be indicated: phospholipids, triglycerides, fatty acids, sterols and glycolipids [29]. In turn, phospholipids are divided into two main classes—sphingophospholipids and glycerophospholipids—depending on the structure of the backbone alcohol (sphingosine or glycerol, respectively). The phospholipid composition of the commercial egg yolk lecithin used in the liposome preparation is illustrated in Figure 2. As seen, the major glycerophospholipids comprise phosphatidylcholine (78.4%); phosphatidylethanolamine (17.6%); phosphatidylinositol (1.2%); phosphatidic acid (1.0%) and phosphatidylserine (1.8%).

The size of resulting liposomes was established using laser diffraction (LD), which is one of the ensemble methods for rapidly measuring the size and distribution of colloidal systems [30]. Laser diffraction particle size analysis showed that the mean particle size of lecithin and cardiolipin liposomes was 583 ± 0.95 nm (*D*_10_ 420 nm, *D*_50_ 583 nm and *D*_90_ 822 nm). Using conventional classifications, the obtained phospholipid liposomes can be attributed to multilamellar vesicles (MLVs) according to their morphology (>500 nm) [31].

In order to elucidate the ability of terpenoid hydrazones **1**–**15** to incorporate into biological membranes, pyrene was selected as a classical fluorescence probe due to its long decay time, its ability to form excimers and irs sensitivity towards the microenvironment [32]. Furthermore, pyrene molecules are predominantly localized at the level of hydrocarbon moieties of the lipid bilayer, which is the incorporation area of free terpenes [33]. The vibrational structure of the pyrene emission spectrum was characterized using five peaks, designated as *I*_1_–*I*_5_ at ~373, 379, 384, 394 and 410 nm, accordingly, which was due to the π → π* transitions. Given that the intensity of peak *I*_1_ is increased in polar solvents, whereas the intensity of band *I*_3_ is dramatically enhanced in hydrophobic environments, the ratio *I*_1_/*I*_3_ (*I*_373_/*I*_384_) was applied to detect the polarity of the pyrene vicinity. Additionally, for monomeric peaks, a broad band appears at longer wavelengths (from 425 to 550 nm, centered at 475 nm) when pyrene molecules in the excited state collide with ground-state pyrene rings. This interaction occurs at a distance ~10 Å between two pyrene forms, leading to the formation of excited-state dimers or “excimers” [34]. Pyrene excimerization clearly depends on the ability of fluorescent probes to laterally diffuse through the membrane. The extent of excimer formation is described mathematically by the excimer/monomer fluorescence intensity ratio (*I*_E_/*I*_M_, *I*_475_/*I*_394_), comparing the fluorescence intensity of the monomer peak at 394 nm (*I*_M_) and the excimer band at 475 nm (*I*_E_). The increase in the number of excimers formed and, consequently, in the *I*_475_/*I*_394_ value indicates the low viscosity of the system, owing to the lateral diffusivity of the pyrene [35].

With a view to estimating the influence of terpenoid hydrazones **1**–**15** on lipid molecular organization, both the excimer-to-monomer (*I*_E_/*I*_M_) and the first-to-third (*I*_1_/*I*_3_) intensity ratios were calculated. The effect of hydrazones on membrane permeability was compared to those of initial terpenoids (verbenone, menthone and carvone) examined under the same experimental conditions.

**Figure 3:**
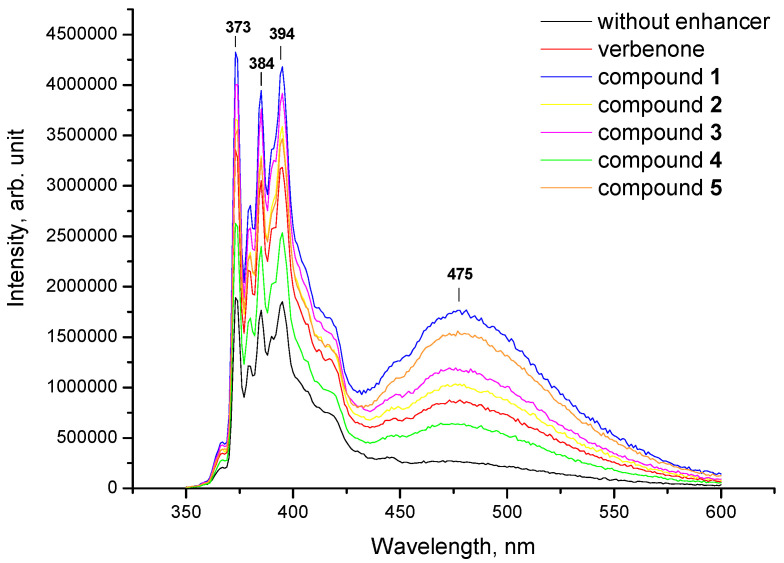
Representative fluorescent emission spectra of pyrene inserted into lecithin liposomes (control, black line) and in the presence of verbenone and its hydrazones **1**–**5**. All measurements were carried out at 25 °C; the excitation wavelength was 338 nm.

The representative emission spectra of pyrene incorporated into the model lecithin liposomes in the presence of verbenone hydrazones (**1**–**5**) are illustrated in Figure 3. As shown, excimer formation was not observed at 475 nm in a sample comprising only lecithin phospholipids and membrane probes. However, the appearance of an excimer peak was detected when verbenone derivatives, along with menthone and carvone hydrazones **6**–**15**, were inserted into liposomal lipids.

The excimer/monomer fluorescence intensity ratio of pyrene (*I*_475_/*I*_394_) has been extensively employed to evaluate the microviscosity of the hydrocarbon interiors of phospholipid membranes [36]. This parameter depends upon the rate of monomer lateral diffusion in lipid bilayers and was found to correlate with membrane fluidity [35,37]. The results of the *I*_E_/*I*_M_ ratio calculation in lecithin liposomes containing verbenone, menthone and carvone, along with their hydrazones **1**–**15**, are summarized in Table 1.

The data reported in Table 1 indicate an increase in the excimer-to-monomer intensity ratio (from 0.179 to 0.440) for the liposome samples involving terpenoids and their derivatives **1**–**15**. In contrast, the *I*_E_/*I*_M_ value for liposomes containing only fluorescent probes and lecithin phospholipids (control) was found to be 0.142 ± 0.004. As seen, *I*_E_/*I*_M_ parameters for the initial terpenoids exceeded the control values two-fold and averaged 0.304. The highest *I*_E_/*I*_M_ ratios were recorded for hydrazones **1**, **3**, **7** and **11**, at 0.426, 0.413, 0.415 and 0.440, respectively. It is worth noting the structure of the above-mentioned compounds that contain the residues of different terpenoids, along with H, Cl or Br atoms in the *para*-position of the benzene ring. Based on fluorescence spectroscopic measurements we may state the influence of verbenone, menthone, carvone and their derivatives **1**–**15** on excimer formation when these compounds were added to lecithin phospholipids. Thus, the incorporation of terpenoid hydrazones into the lecithin liposomes contributes to a decrease in lipid microviscosity; disruption of their packing density, resulting in pyrene displacement; and, consequently, excimer formation.

Since pyrene is an environmentally-sensitive fluorophore, it might be effectively used in order to estimate the micropolarity of the pyrene’s vicinity by calculating the ratio of the first (373 nm) to the third (384 nm) vibronic band (*I*_1_/*I*_3_). The results of the determination of the *I*_1_/*I*_3_ values in lecithin liposomes with inserted mono-/bicycle terpenoids and hydrazones **1**–**15** are presented in Table 2.

For lecithin liposome samples containing only incorporated fluorescence probes, the *I*_1_/*I*_3_ parameter was shown to be 1.067 ± 0.008. The addition of initial terpenoids and hydrazones **1**–**15** to phospholipids during liposome preparation caused a slight change in the *I*_1_/*I*_3_ value from 0.956 to 1.134. Thus, an increase in the polarity of the fluorophore microenvironment was observed when adding initial terpenoids and their derivatives **1**, **2**, **4**, **6**, **10** to lecithin lipids, indicating no significant structure–property relationships.

**Figure 4:**
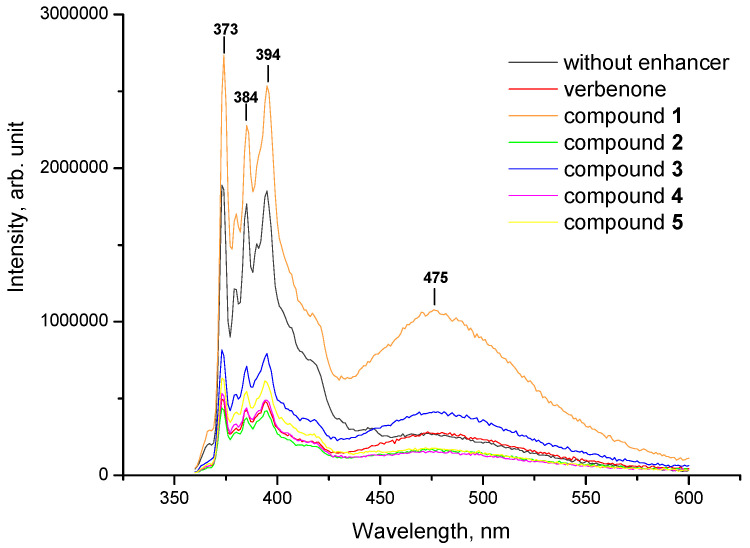
Representative fluorescent emission spectra of pyrene inserted into cardiolipin liposomes (control, black line) and in the presence of verbenone and its hydrazones **1**–**5**. All measurements were carried out at 25 °C; the excitation wavelength was 338 nm.

**Figure 5:**
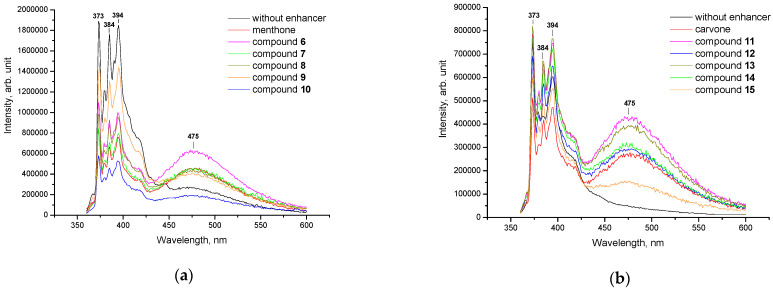
Representative fluorescent emission spectra of pyrene inserted into cardiolipin liposomes (control, black line) and in the presence of menthone **6**–**10**, (**a**) and carvone **11**–**15**, (**b**) hydrazones. All measurements were carried out at 25 °C; the excitation wavelength was 338 nm.

Additionally, the impact of terpenoids and their hydrazones on the molecular organization of the lipid matrix was investigated using model liposomes based on cardiolipin. Figure 4 and Figure 5 illustrate the representative emission spectra of pyrene inserted into the cardiolipin liposomes comprising verbenone hydrazones (**1**–**5**), menthone (**6**–**10**) and carvone (**11**–**15**) derivatives. As seen, an unstructured band appears at longer wavelengths (ranging from 425 to 550 nm) with a peak at 475 nm when verbenone hydrazones, along with menthone and carvone derivatives **6**–**15**, were incorporated into cardiolipin liposomes.

The inclusion of terpenoids and hydrazones **1**–**15** into cardiolipin liposomes was followed by an increase in pyrene fluorescence in the field of excimer formation (450–550 nm) compared to controls (Table 3). As highlighted, consistently high values of the *I*_E_/*I*_M_ parameter are typical for initial terpenoids, at 0.574, 0.599 and 0.570 for verbenone, menthone and carvone, accordingly. Among hydrazones, the greatest impact on pyrene lateral diffusion was revealed for compounds **3**, **6**, **7**, **11** and **13** with *I*_E_/*I*_M_ ratios 0.529, 0.621, 0.571, 0.570 and 0.511, respectively. By analyzing the structure–property relationship, the following substituents in hydrazone structures should be emphasized: H, Cl or Br atoms in the *para*-position of the benzene ring, which is in accordance with the experiment involving lecithin liposomes. Hence, the incorporation of terpenoid hydrazones into cardiolipin liposomes leads to the enhancement of pyrene lateral mobility, indicating the excimerization of the fluorescence probe.

The *I*_1_/*I*_3_ ratio, which indicates an increase in the polarity of the pyrene microenvironment has been also calculated for cardiolipin liposomes. As shown in Table 4, a significant increase in the *I*_1_/*I*_3_ parameter was predominantly observed after the incorporation of hydrazones with bulky -C(CH_3_)_3_ and -O-C_6_H_5_ groups into lipid membranes. This growth of the *I*_1_/*I*_3_ value demonstrates an increase in the polarity of the fluorophore microenvironment, which might be caused by the appearance of hydrophilic clusters in cardiolipin lipid layers.

Thus, based on fluorescence analysis, we may suggest a reduction in microviscosity and, consequently, a decrease in the packing density of lecithin and cardiolipin lipids, as the major mechanism of action for terpenoid hydrazones with H, Cl or Br atoms in the *para*-position of the benzene ring. Hydrazones containing bulky -C(CH_3_)_3_ and -O-C_6_H_5_ groups were also found to increase the membrane polarity via the appearance of hydrophilic clusters or via the penetration of water molecules into the lipid layers of cardiolipin liposomes.

### 2.2. FT-IR Spectroscopy Investigation

Along with fluorescence probe studies, Fourier-transform infrared spectroscopy (FT-IR) refers to a significant technique for examination the molecular organization of an artificial lipid membrane or lipid isolated from the stratum corneum [38,39]. In our research, the FT-IR spectroscopy method was applied to suggest the mechanism of interaction between lipids isolated from rat strata cornea and terpenoid hydrazones. This measurement was based on the estimation of hydrogen-bonding interactions between SC lipids and terpenoid derivatives **1**–**15**. In this regard, the changes in the absorbance intensity of functional groups of SC constituents were analyzed.

The general structure of the SC involves an array of keratinized cells embedded in a lipid matrix [40]. Thus, the lipids of the stratum corneum consist of two groups: freely extractable intercellular lipids and covalently bound lipids of the corneocyte membrane. SC permeability is identified precisely by intercellular lipids belonging to the following classes: ceramides (Cer), cholesterol (Chol) and long-chain free fatty acids (FFA) that are extracted by means of a chloroform:methanol system [41,42]. Recently, we determined the lipid composition extracted from the SC via a mixture of aforementioned solvents and defined cholesterol, cholesterol oleate, fatty acids, triglycerides and ceramides as major components of the SC intercellular space [15]. Bearing in mind the chemical structures of the designated compounds, the most significant ones for FT-IR analysis were the absorption bands of functional groups that form a network of hydrogen bonds in the lipid matrix, namely, OH and C=O groups (see Supplementary Materials).

Three main peaks were observed in the FT-IR spectrum of pure lipids isolated from SCs: a band at 3393 cm^−1^ corresponding to stretching vibrations of the associated OH groups; two absorption bands at 1737 cm^−1^ and 1656 cm^−1^ of the carbonyl C=O group associated with the monomeric state (1737 cm^−1^) and carboxylic acid dimmers (1656 cm^−1^). The values of peak intensity at 3393, 1737 and 1656 cm^−1^ for samples of SC lipids comprising hydrazones **1**–**15** along with initial terpenoids (verbenone, menthone and carvone) are summarized in Table 5. The intensity of the abovementioned bands was expressed as the percentage of incident light transmission (T), which is inversely proportional to the peak intensity. Strong hydrogen-bonding interactions between the hydroxyl groups of SC lipids was observed as a broad band at 3393 cm^−1^ with an intensity of transmission of 78%. A decrease in the intensity of OH stretching vibrations was recorded after the addition of terpenoids and their derivatives **1**–**15**; this phenomenon is associated with the disruption of hydrogen bonding in the SC lipid matrix. The most pronounced influence was revealed by incorporating hydrazones with H, Cl or Br atoms in the *para*-position of the benzene ring into SC lipids (with an average percentage of transmission of 15%–29%). The same trend was typical for the C=O stretching frequency of carboxylic acid dimmers (1656 cm^−1^)—there was a reduction in the intensity of this band when mixing lipids with terpenoid derivatives **1**–**3**, **6**–**8**, **11**–**13**, whereas hydrazones containing bulky -C(CH_3_)_3_ and -O-C_6_H_5_ groups exhibited no effect in lipid liquefaction. Notably, both pure terpenoids and their hydrazones displayed a slight impact on C=O group vibrations related to its monomeric form (1737 cm^−1^). Hence, the disruption of the hydrogen-bonded network formed by polar groups of SC lipids might be suggested as a mechanism of action for terpenoid hydrazones **1**–**15**.

In this manner, the results of the investigation of hydrazones as biomembrane penetration enhancers using fluorescence probe studies and FT-IR spectroscopy indicated the increased permeability of these compounds across membranes. These data further substantiate the high pharmacological effect of terpenoid derivatives and fully correlate with previously published data [21,22,23] regarding the analgesic and anticonvulsant activity of the abovementioned compounds. According to our study, the higher antinociceptive and antiseizure potency after topical delivery and oral administration, respectively, was displayed by terpenoid derivatives with H, Cl or Br atoms in the *para*-position of the benzene ring. This work is an example of compounds’ designs simultaneously influencing their different pharmacological targets and enhancing their self-permeability.

## 3. Materials and Methods

### 3.1. General

Egg yolk lecithin and cardiolipin from the bovine heart were obtained from Biolek (Kharkov, Ukraine). Pyrene and trypsin (from the bovine pancreas) were purchased from Merck (Darmstadt, Germany). All organic solvents and other chemicals used were of analytical grade. Terpenoid hydrazones **1**–**15** were synthesized and fully characterized in our previous studies [21,22,23]. Pure terpenoids were used as obtained from their commercial supplier: (–)-verbenone, (–)-menthone and (–)-carvone (TCI, Philadelphia, PA, USA).

### 3.2. Liposome Preparation

Liposomes based on lecithin or cardiolipin were prepared using the thin-film evaporation method. For this purpose, solutions of phospholipids (lecithin or cardiolipin, 0.02 mol/L), pure terpenoids or their hydrazones **1**–**15** (0.002 mol/L) and pyrene (0.0002 mol/L) were prepared in chloroform. One milliliter of each above-mentioned solution was taken and placed in a round-bottom flask. Then, the solvent was removed via slow evaporation under a vacuum at 40 °C. The dried mixture was resuspended in 10 mL of deionized water and vigorously stirred for 10 min. The resulting emulsion was then sonicated for 10 min at a frequency of 22 kHz. All liposomes were freshly prepared on the day of the experiment.

### 3.3. Determination of Liposome Size Distribution

The particle size distributions of both lecithin and cardiolipin liposomes were determined via laser diffraction using a Mastersizer 3000 equipped with a Hydro SM dispersion unit (Malvern Instruments, Malvern, UK). The stirrer speed was set at 2000 rpm; distilled water was used as a dispersant. The obscuration value was in the range of 1–5% for each analysis. Results are expressed as volume median diameters *D*_10_, *D*_50_ and *D*_90_.

### 3.4. Fluorescence Measurements

The steady-state fluorescence emission spectra of liposomes solutions containing pyrene were recorded on a Horiba Jobin-Yvon Fluorog-FL 3-22 spectrophotometer, equipped with a 450 W Xe lamp with the use of 1 cm path-length quartz cuvettes. The excitation wavelength for all samples containing pyrene was 338 nm. The slit width of both excitation and emission was set at 2 nm. The fluorescence intensity ratios of the first to third vibronic bands (*I*_1_/*I*_3_) were determined at 373 nm and 384 nm, respectively. The excimer/monomer emission intensity (*I*_E_/*I*_M_) ratio was calculated by measuring the relative intensities of pyrene excimer and monomer forms at 394 nm and 475 nm, respectively.

### 3.5. Experimental Animals

Skin samples were collected from male Wistar rats (150–180 g). All animals were kept under a 12 h light regime in a standard animal facility with free access to water and food, in compliance with the European Convention for the Protection of Vertebrate Animals Used for Experimental and Other Specific Purposes (Strasbourg, 1986), ARRIVE guidelines and the principles of the National Ukrainian Bioethics Congress (Kyiv, 2003). All animals were purchased from Odessa National Medical University, Ukraine. The Animal Ethics Committee (agreement No. 6/2021) of Odessa National Polytechnic University (Ukraine) approved the study.

### 3.6. Isolation of Stratum Corneum

Skin sites were selected from large, uniform body areas of the male Wistar rats. The rats were sacrificed with the inhalation of an excess of chloroform, followed by shaving and surgical removal of the abdominal and back regions. After the removal of subcutaneous fat tissue using a scalpel, the SC was separated from the epidermis by incubating it in trypsin solution (0.15% in PBS buffer, pH 7.4) for 24 h at 4 °C and thereafter for 4 h at 37 °C. Then, the SC was mechanically separated and its trypsinazation was terminated via the addition of trypsin inhibitor solution, with subsequent deionized water washes and drying.

### 3.7. Extraction of SC Lipids

SC sheets were homogenized, dipped into chloroform:methanol (2:1) solution and kept in the dark for 72 h. Then the extract was separated, washed twice with distilled water and the lower organic lipid-containing layer was evaporated to dryness under a nitrogen atmosphere below 40 °C.

### 3.8. FT-IR Spectroscopy 

FT-IR spectra were recorded on a Frontier FT-IR spectrometer (Perkin-Elmer, Hopkinton, MA, USA). The samples for FT-IR studies were prepared by dissolving the lipids isolated from SCs in carbon tetrachloride (CCl_4_), followed by addition of terpenoid hydrazones **1**–**15** (10% relative to lipids’ mass). FT-IR spectra were measured for films obtained using a technique of slow solvent evaporation directly from undercover in a nitrogen atmosphere. All FT-IR investigations were performed at room temperature (25 °C).

### 3.9. Statistical Analysis 

All results are expressed as mean ± standard error mean (SEM). One-way analysis of variance (ANOVA) was used to determine the statistical significance of the results, followed by Tukey’s post hoc comparison. *p* < 0.05 was considered significant.

## 4. Conclusions

In this paper, we confirmed the impact of terpenoid hydrazones containing residues of *para*-substituted phenoxyacetic acid on the molecular organization of the lipid matrix. Fluorescence probe analysis with the use of lecithin and cardiolipin liposomes suggested that terpenoid derivatives decrease phospholipid microviscosity and disrupt their packing density. Considering the potential application of compounds **1**–**15** as topical agents, their influence on lipids isolated from rat stratum corneum was investigated by FT-IR spectroscopy. The disruption of the hydrogen-bonded network formed by polar groups of stratum corneum lipids was proposed as a mechanism of action for terpenoid hydrazones **1**–**15**. Hydrazones containing the residues of verbenone, carvone and menthone, along with H, Cl or Br atoms in the *para*-position of the benzene ring, were found to achieve a higher effect as biomembrane penetration enhancers; these results further substantiate the high pharmacological effects observed for terpenoid derivatives and correlate with the previously obtained data on the anticonvulsant and analgesic activity of the abovementioned compounds. Thus, the influence of terpenoid hydrazones on the molecular packing of lipids substantiates the feasibility of their use both after oral administration and in transdermal delivery in vivo.

## Figures and Tables

**Table 1:** Pyrene excimer-to-monomer fluorescence intensity ratio *I*_E_/*I*_M_ in lecithin liposomes with hydrazones **1**–**15** incorporated into the lipid membrane (*n* = 3).

Compound	*I*_E_/*I*_M_	Compound	*I*_E_/*I*_M_
Verbenone	0.269 ± 0.002	**8**	0.311 ± 0.010
Menthone	0.353 ± 0.008	**9**	0.264 ± 0.003
Carvone	0.291 ± 0.003	**10**	0.272 ± 0.005
**1**	0.426 ± 0.004	**11**	0.440 ± 0.009
**2**	0.293 ± 0.008	**12**	0.220 ± 0.011
**3**	0.413 ± 0.004	**13**	0.278 ± 0.004
**4**	0.248 ± 0.005	**14**	0.294 ± 0.008
**5**	0.305 ± 0.003	**15**	0.179 ± 0.004
**6**	0.352 ± 0.002	Control	0.142 ± 0.004
**7**	0.415 ± 0.009		

For all groups *p* ˂ 0.01 compared to control.

**Table 2:** The ratio of pyrene monomer fluorescence intensity *I*_1_/*I*_3_ in lecithin liposomes with hydrazones **1**–**15** incorporated into the lipid membrane (*n* = 3).

Compound	*I*_1_/*I*_3_	Compound	*I*_1_/*I*_3_
Verbenone	1.105 ± 0.008 *	**8**	1.072 ± 0.012
Menthone	1.118 ± 0.004 **	**9**	1.086 ± 0.010
Carvone	1.134 ± 0.002 **	**10**	1.112 ± 0.006 **
**1**	1.104 ± 0.007 *	**11**	1.082 ± 0.011
**2**	1.113 ± 0.003 **	**12**	0.956 ± 0.008
**3**	1.072 ± 0.002	**13**	0.991 ± 0.003
**4**	1.101 ± 0.002 *	**14**	1.016 ± 0.004
**5**	1.095 ± 0.009	**15**	1.086 ± 0.009
**6**	1.122 ± 0.005 **	Control	1.067 ± 0.008
**7**	1.084 ± 0.011		

* *p* ˂ 0.05, ** *p* ˂ 0.01 compared to control.

**Table 3:** Pyrene excimer-to-monomer fluorescence intensity ratio *I*_E_/*I*_M_ in cardiolipin liposomes with hydrazones **1**–**15** incorporated into the lipid membrane (*n* = 3).

Compound	*I*_E_/*I*_M_	Compound	*I*_E_/*I*_M_
Verbenone	0.574 ± 0.004	**8**	0.448 ± 0.010
Menthone	0.599 ± 0.003	**9**	0.289 ± 0.002
Carvone	0.570 ± 0.006	**10**	0.374 ± 0.004
**1**	0.426 ± 0.002	**11**	0.570 ± 0.005
**2**	0.384 ± 0.004	**12**	0.444 ± 0.009
**3**	0.529 ± 0.009	**13**	0.511 ± 0.011
**4**	0.315 ± 0.011	**14**	0.437 ± 0.008
**5**	0.280 ± 0.009	**15**	0.287 ± 0.006
**6**	0.621 ± 0.006	Control	0.146 ± 0.004
**7**	0.571 ± 0.004		

For all groups *p* ˂ 0.01 compared to control.

**Table 4:** The ratio of pyrene monomer fluorescence intensity *I*_1_/*I*_3_ in cardiolipin liposomes with hydrazones **1**–**15** incorporated into the lipid membrane (*n* = 3).

Compound	*I*_1_/*I*_3_	Compound	*I*_1_/*I*_3_
Verbenone	1.096 ± 0.010	**8**	1.122 ± 0.011 *
Menthone	1.260 ± 0.009 **	**9**	1.143 ± 0.008 **
Carvone	1.236 ± 0.002 **	**10**	1.262 ± 0.004 **
**1**	1.026 ± 0.011	**11**	1.197 ± 0.005 **
**2**	1.217 ± 0.008 **	**12**	1.212 ± 0.010 **
**3**	1.157 ± 0.004 **	**13**	1.217 ± 0.009 **
**4**	1.248 ± 0.008 **	**14**	1.196 ± 0.007 **
**5**	1.155 ± 0.006 **	**15**	1.232 ± 0.006 **
**6**	1.181 ± 0.007 **	Control	1.081 ± 0.002
**7**	1.163 ± 0.010 **		

* *p* ˂ 0.05, ** *p* ˂ 0.01 compared to control.

**Table 5:** The intensity of absorption bands in the FT-IR spectra of samples containing hydrazones **1**–**15** and lipids isolated from the SC.

Compound	Intensity of Band, % of Transmission (T)/Absorbance		
	3393 cm^−1^	1737 cm^−1^	1656 cm^−1^
Verbenone	31.5/0.502	68.6/0.164	30.9/0.510
Menthone	27.7/0.558	62.3/0.206	18.9/0.724
Carvone	26.2/0.582	64.4/0.191	24.2/0.616
**1**	16.2/0.790	54.2/0.266	21.9/0.660
**2**	21.3/0.672	70.1/0.154	27.6/0.559
**3**	27.6/0.559	76.8/0.115	30.7/0.513
**4**	40.6/0.391	93.2/0.031	56.2/0.250
**5**	30.0/0.523	40.2/0.396	20.3/0.693
**6**	15.6/0.807	36.1/0.442	25.0/0.602
**7**	25.6/0.592	64.6/0.190	27.8/0.556
**8**	28.0/0.553	57.3/0.242	24.9/0.604
**9**	44.1/0.356	76.8/0.115	41.6/0.381
**10**	47.9/0.320	89.1/0.050	45.7/0.340
**11**	16.0/0.796	48.2/0.317	24.3/0.614
**12**	22.8/0.642	56.7/0.246	29.1/0.536
**13**	29.8/0.526	62.8/0.202	27.5/0.561
**14**	46.2/0.335	72.8/0.138	49.1/0.309
**15**	51.3/0.290	66.9/0.175	42.7/0.370
Control	78.8/0.103	44.1/0.356	42.2/0.375

## Data Availability

The data presented in this study are contained within the article.

## Supplementary Materials

The following are available online. Figures S1–S19, FT-IR spectra of pure stratum corneum (SC) lipids and samples containing hydrazones **1**–**15**, along with lipids isolated from SCs.

## Sample Availability

Samples are available from the authors.

## References

[1] Aungst B.J. (2012). Absorption enhancers: Applications and advances. AAPS J..

[2] Prausnitz M., Langer R. (2008). Transdermal drug delivery. Nat. Biotechnol..

[3] N’Da D.D. (2014). Prodrug strategies for enhancing the percutaneous absorption of drugs. Molecules.

[4] Wang J.J., Sung K.C., Huang J.F., Yeh C.H., Fang J.Y. (2007). Ester prodrugs of morphine improve transdermal drug delivery: A mechanistic study. J. Pharm. Pharmacol..

[5] Stinchcomb A.I., Swaan P.W., Ekabo O., Harris K.K., Browe J., Hammell D.C., Cooperman T.A., Pearsall M. (2002). Straight-chain naltrexone ester prodrugs: Diffusion and concurrent esterase biotransformation in human skin. J. Pharm. Sci..

[6] Qandil A., Al-Nabulsi S., Al-Taani B., Tashtoush B. (2008). Synthesis of piperazinylalkyl ester prodrugs of ketorolac and their *in vitro* evaluation for transdermal delivery. Drug Dev. Ind. Pharm..

[7] Kiptoo P.K., Paudel K.S., Hammell D.C., Pinninti R.R., Chen J., Crooks P.A., Stinchcomb A.L. (2009). Transdermal delivery of bupropion and its active metabolite, hydroxybupropion: A prodrug strategy as an alternative approach. J. Pharm. Sci..

[8] Kerr D., Roberts W., Tebbett I., Sloan K.B. (1998). 7-Alkylcarbonyloxymethyl prodrugs of theophylline: Topical delivery of theophylline. Int. J. Pharm..

[9] Morris A.P., Brain K.R., Heard C.M. (2009). Skin permeation and ex vivo skin metabolism of O-acyl haloperidol ester prodrugs. Int. J. Pharm..

[10] Chen J., Jiang Q.-D., Chai Y.-P., Zhang H., Peng P., Yang X.-X. (2016). Natural terpenes as penetration enhancers for transdermal drug delivery. Molecules.

[11] Cox-Georgian D., Ramadoss N., Dona C., Basu C., Joshee N., Dhekney S., Parajuli P. (2019). Therapeutic and medicinal uses of terpenes. Medicinal Plants.

[12] Nóbrega de Almeida R., Agra M.d.F., Negromonte Souto Maior F., De Sousa D.P. (2011). Essential oils and their constituents: Anticonvulsant activity. Molecules.

[13] Wang C.-Y., Chen Y.-W., Hou C.-Y. (2019). Antioxidant and antibacterial activity of seven predominant terpenoids. Int. J. Food Prop..

[14] De Sousa D.P. (2011). Analgesic-like activity of essential oils constituents. Molecules.

[15] Nesterkina M., Smola S., Kravchenko I. (2019). Effect of esters based on terpenoids and GABA on fluidity of phospholipid membranes. J. Liposome Res..

[16] Nesterkina M., Kravchenko I. (2016). Synthesis and pharmacological properties of novel esters based on monocyclic terpenes and GABA. Pharmaceuticals.

[17] Premkumar L.S. (2014). Transient receptor potential channels as targets for phytochemicals. ACS Chem. Neurosci..

[18] Manayi A., Nabavi S.M., Daglia M., Jafari S. (2016). Natural terpenoids as a promising source for modulation of GABAergic system and treatment of neurological diseases. Pharmacol. Rep..

[19] Pages N., Maurois P., Bac P., Eynde J.J.V., Tamariz J., Labarrios F., Chamorro G., Vamecq J. (2011). The α-asarone/clofibrate hybrid compound, 2-methoxy-4-(2-propenyl)phenoxyacetic acid (MPPA), is endowed with neuroprotective and anticonvulsant potentialities. Biomed. Aging Pathol..

[20] Turan-Zitouni G., Yurttaş L., Kaplancıklı Z.A., Can Ö.D., Özkay Ü.D. (2015). Synthesis and anti-nociceptive, anti-inflammatory activities of new aroyl propionic acid derivatives including N-acylhydrazone motif. Med. Chem. Res..

[21] Nesterkina M., Barbalat D., Zheltvay I., Rakipov I., Atakay M., Salih B., Kravchenko I. (2019). (2*S*,5*R*)-2-Isopropyl-5-methylcyclohexanone hydrazones. Molbank.

[22] Nesterkina M., Barbalat D., Kravchenko I. (2020). Design, synthesis and pharmacological profile of (−)-verbenone hydrazones. Open Chem..

[23] Nesterkina M., Barbalat D., Konovalova I., Shishkina S., Atakay M., Salih B., Kravchenko I. (2021). Novel (‒)-carvone derivatives as potential anticonvulsant and analgesic agents. Nat. Prod. Res..

[24] Ahad A., Aqil M., Ali A. (2016). The application of anethole, menthone, and eugenol in transdermal penetration of valsartan: Enhancement and mechanistic investigation. Pharm. Biol..

[25] Suhonen M., Li S.K., Higuchi W.I., Herron J.N. (2008). A liposome permeability model for stratum corneum lipid bilayers based on commercial lipids. J. Pharm. Sci..

[26] Reddy A.S., Zhang S. (2013). Polypharmacology: Drug discovery for the future. Expert. Rev. Clin. Pharmacol..

[27] Masnoon N., Shakib S., Kalisch-Ellett L., Caughey G.E. (2017). What is polypharmacy? A systematic review of definitions. BMC Geriatr..

[28] Routledge S.J., Linney J.A., Goddard A.D. (2019). Liposomes as models for membrane integrity. Biochem. Soc. Trans..

[29] Palacios L.E., Wang T. (2005). Egg-yolk lipid fractionation and lecithin characterization. J. Amer. Oil Chem. Soc..

[30] Pei Y., Hinchliffe B.A., Minelli C. (2021). Measurement of the size distribution of multimodal colloidal systems by laser diffraction. ACS Omega.

[31] Maja L., Željko K., Mateja P. (2020). Sustainable technologies for liposome preparation. J. Supercrit. Fluids.

[32] Bains G., Patel A.B., Narayanaswami V. (2011). Pyrene: A probe to study protein conformation and conformational changes. Molecules.

[33] Sapra B., Jain S., Tiwary A.K. (2008). Percutaneous permeation enhancement by terpenes: Mechanistic view. AAPS J..

[34] Bains G.K., Kim S.H., Sorin E.J., Narayanaswami V. (2012). The extent of pyrene excimer fluorescence emission is a reflector of distance and flexibility: Analysis of the segment linking the LDL receptor-binding and tetramerization domains of apolipoprotein E3. Biochemistry.

[35] Ando Y., Asano Y., Le Grimellec C. (1995). Pyrene fluorescence: A potential tool for estimation of short-range lateral mobility in membranes of living renal epithelial cells. Renal Physiol. Biochem..

[36] Melnick R.L., Haspel H.C., Goldenberg M., Greenbaum L.M., Weinstein S. (1981). Use of fluorescent probes that form intramolecular excimers to monitor structural changes in model and biological membranes. Biophys. J..

[37] Chaudhuri A., Haldar S., Chattopadhyay A. (2009). Organization and dynamics in micellar structural transition monitored by pyrene fluorescence. Biochem. Biophys. Res. Commun..

[38] Lewis N.A.H.R., McElhaney R.N. (2013). Membrane lipid phase transitions and phase organization studied by Fourier transform infrared spectroscopy. Biochim Biophys. Acta Biomembr..

[39] Blume A. (1996). Properties of lipid vesicles: FT-IR spectroscopy and fluorescence probe studies. Curr. Opin. Colloid Interface Sci..

[40] Wertz P.W. (2018). Lipids and the permeability and antimicrobial barriers of the skin. J. Lipids.

[41] Mueller J., Schroeter A., Steitz R., Trapp M., Neubert R.H. (2016). Preparation of a new oligolamellar stratum corneum lipid model. Langmuir.

[42] Gooris G.S., Bouwstra J.A. (2007). Infrared spectroscopic study of stratum corneum model membranes prepared from human ceramides, cholesterol, and fatty acids. Biophys. J..

