# Supplementary material for: Terpenoid Hydrazones as Biomembrane Penetration Enhancers: FT-IR Spectroscopy and Fluorescence Probe Studies
Source: Molecules. 2021 Dec 29;27(1):206. doi: 10.3390/molecules27010206 (PMC8746376; doi:10.3390/molecules27010206)
Supplement: Supplementary file 1 [file molecules-27-00206-s001.zip › molecules-1510356-supplementary.pdf]

Supplementary Material: Terpenoid Hydrazones as Biomembrane Penetration Enhancers: FT-IR Spectroscopy and Fluorescence Probe Studies

Mariia Nesterkina, Serhii Smola, Nataliya Rusakova, and Iryna Kravchenko

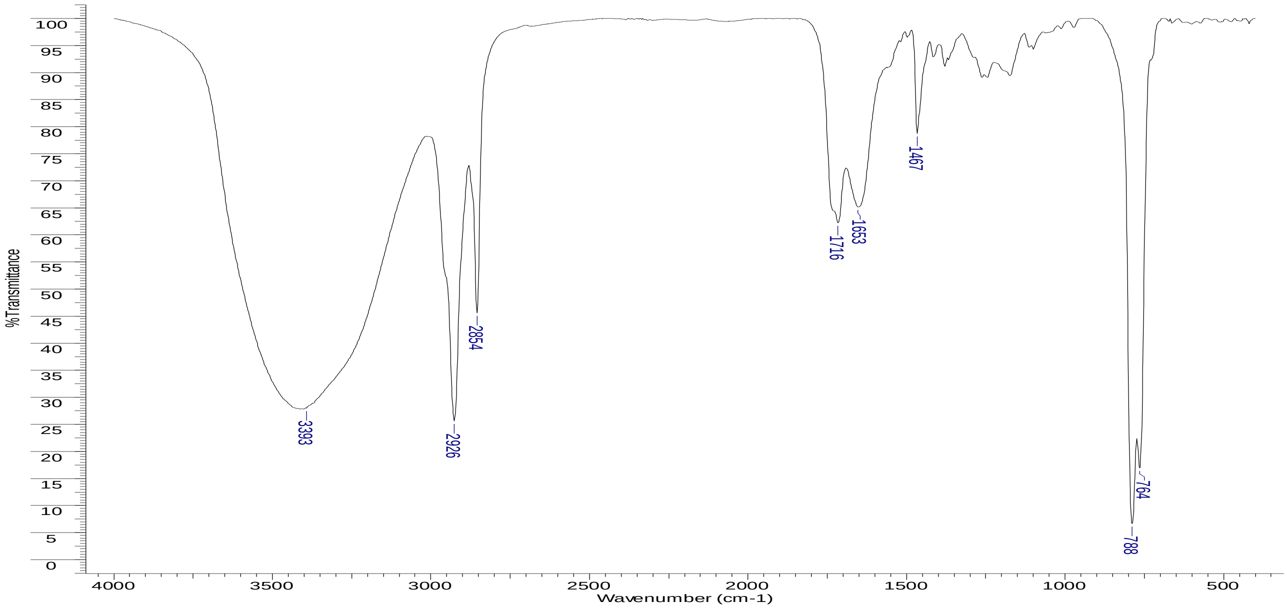

Figure S1. FT-IR spectrum of pure lipids isolated from stratum corneum (SC).

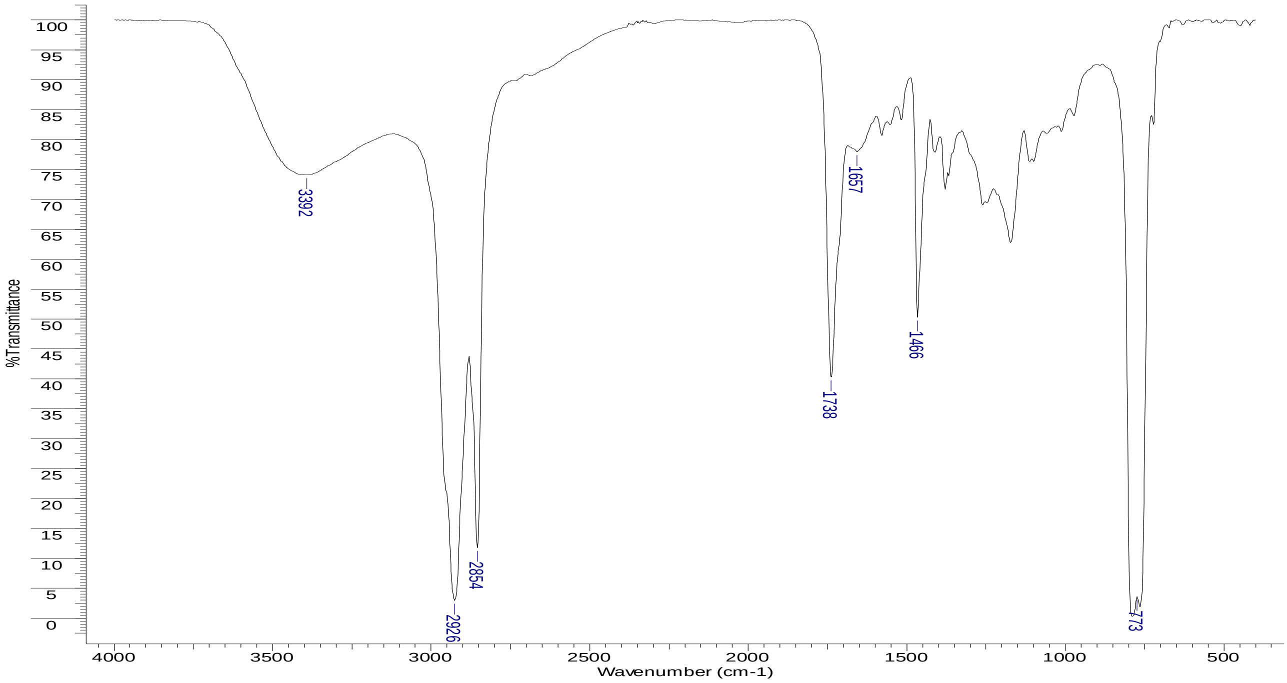

Figure S2. FT-IR spectrum of sample containing verbenone and lipids isolated from SC.

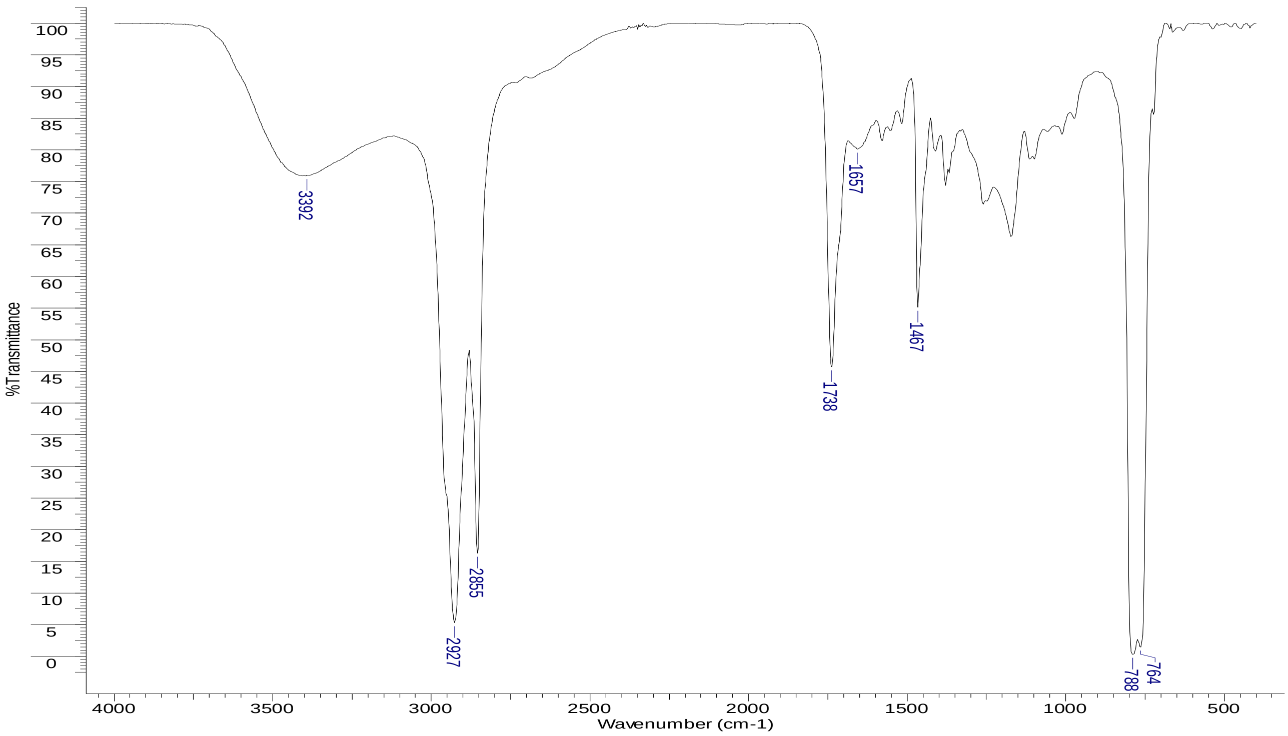

Figure S3. FT-IR spectrum of sample containing menthone and lipids isolated from SC.

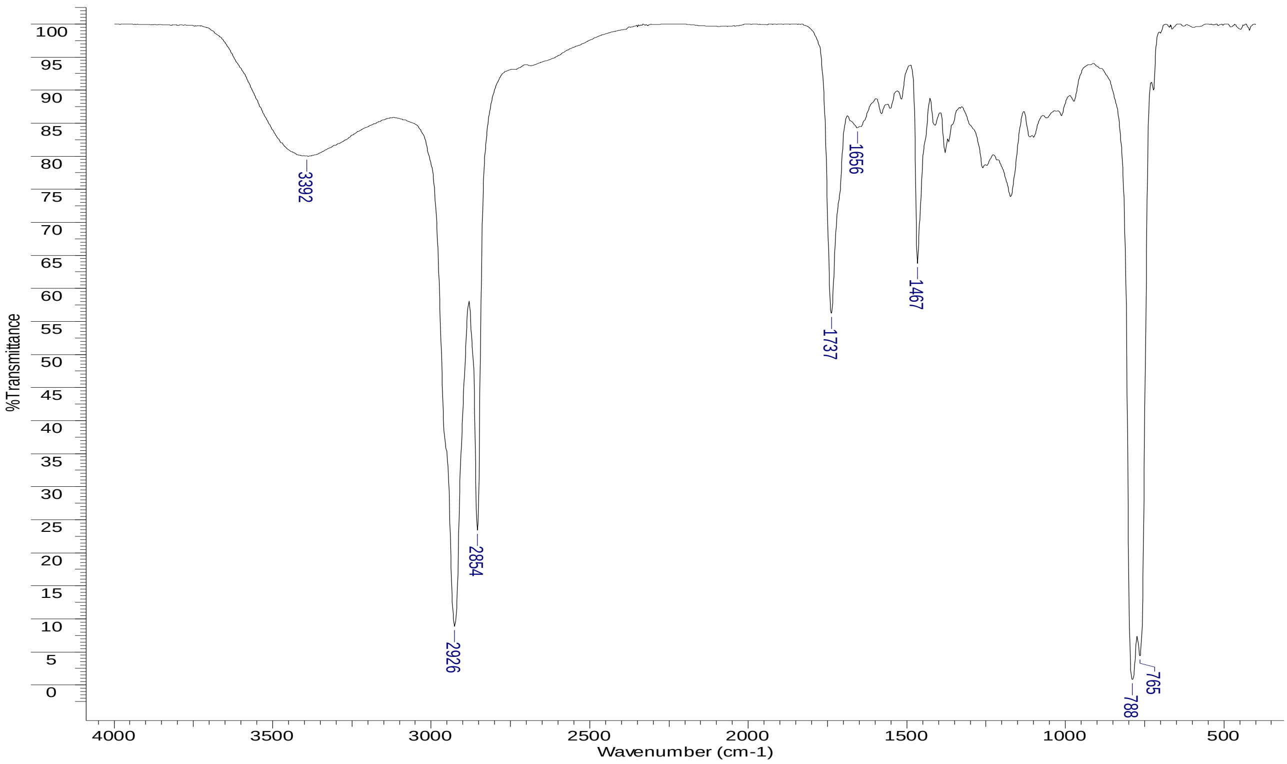

Figure S4. FT-IR spectrum of sample containing carvone and lipids isolated from SC.

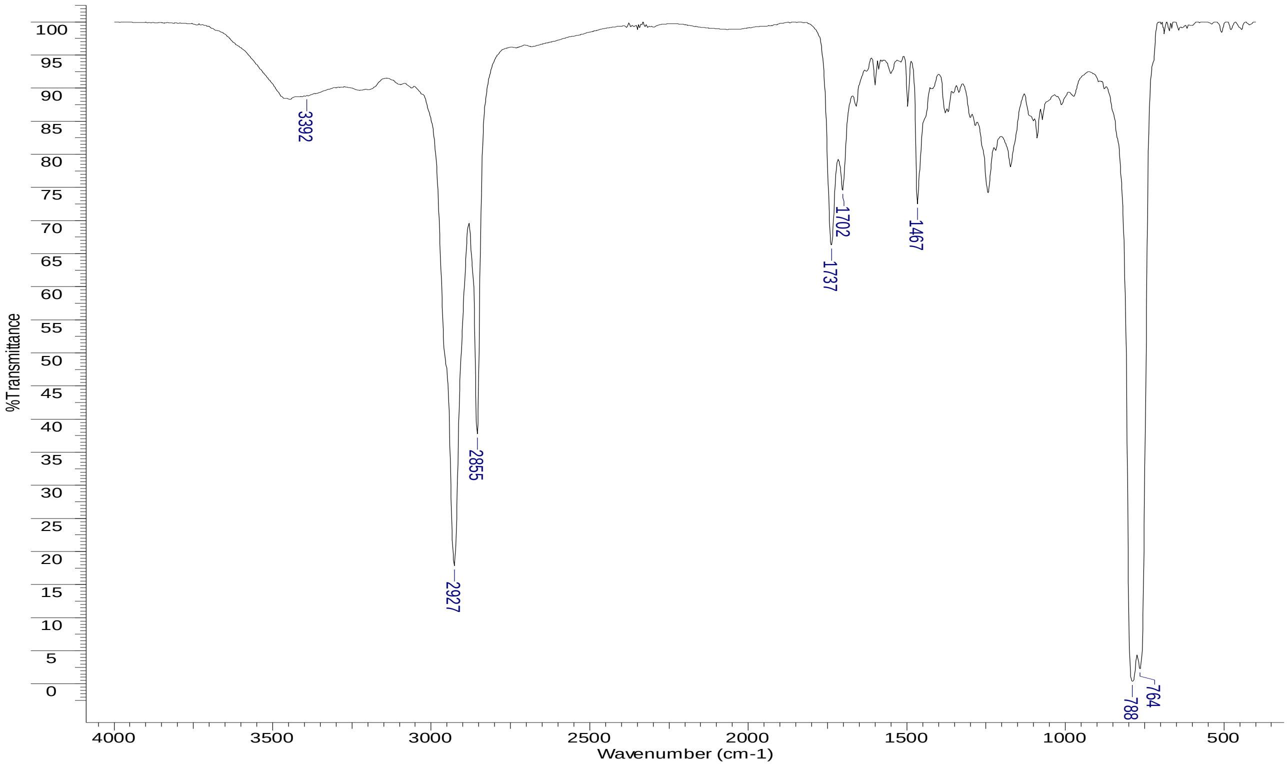

Figure S5. FT-IR spectrum of sample containing verbenone hydrazone **1** and lipids isolated from SC.

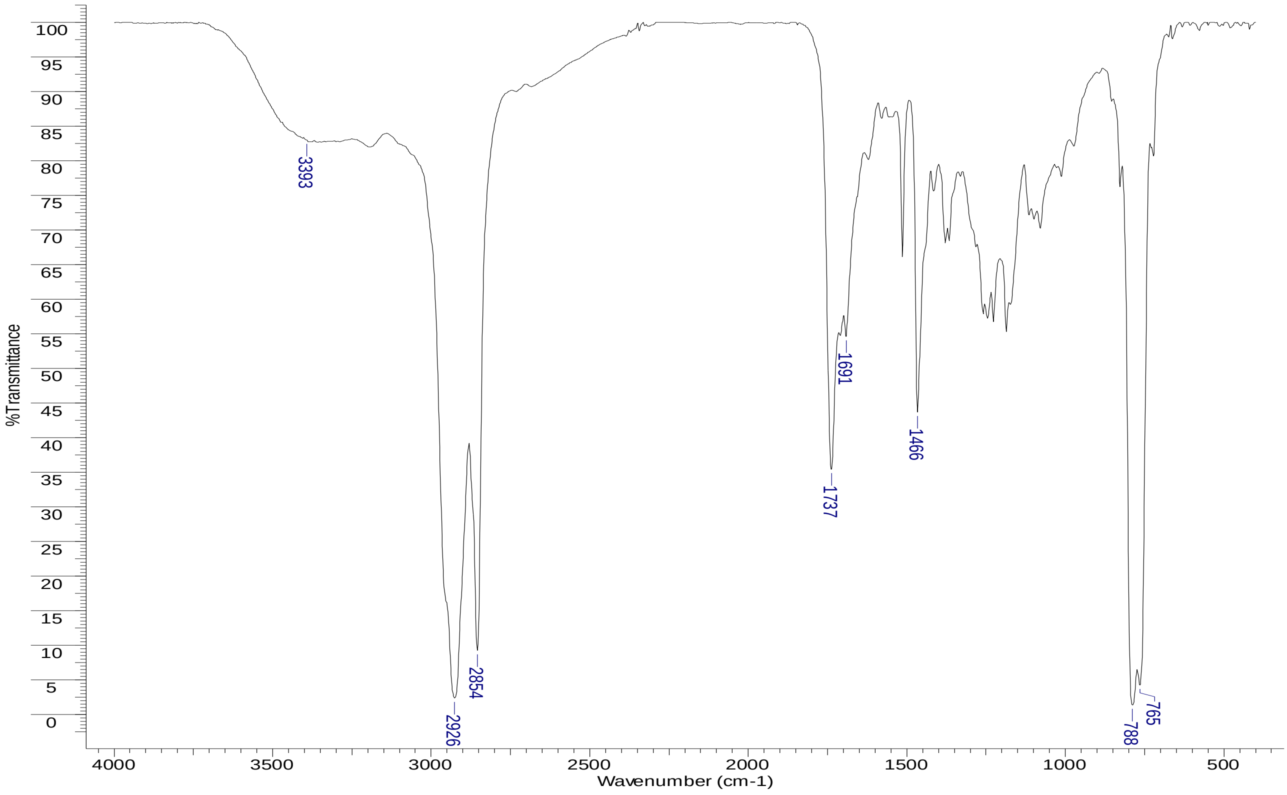

Figure S6. FT-IR spectrum of sample containing verbenone hydrazone **2** and lipids isolated from SC.

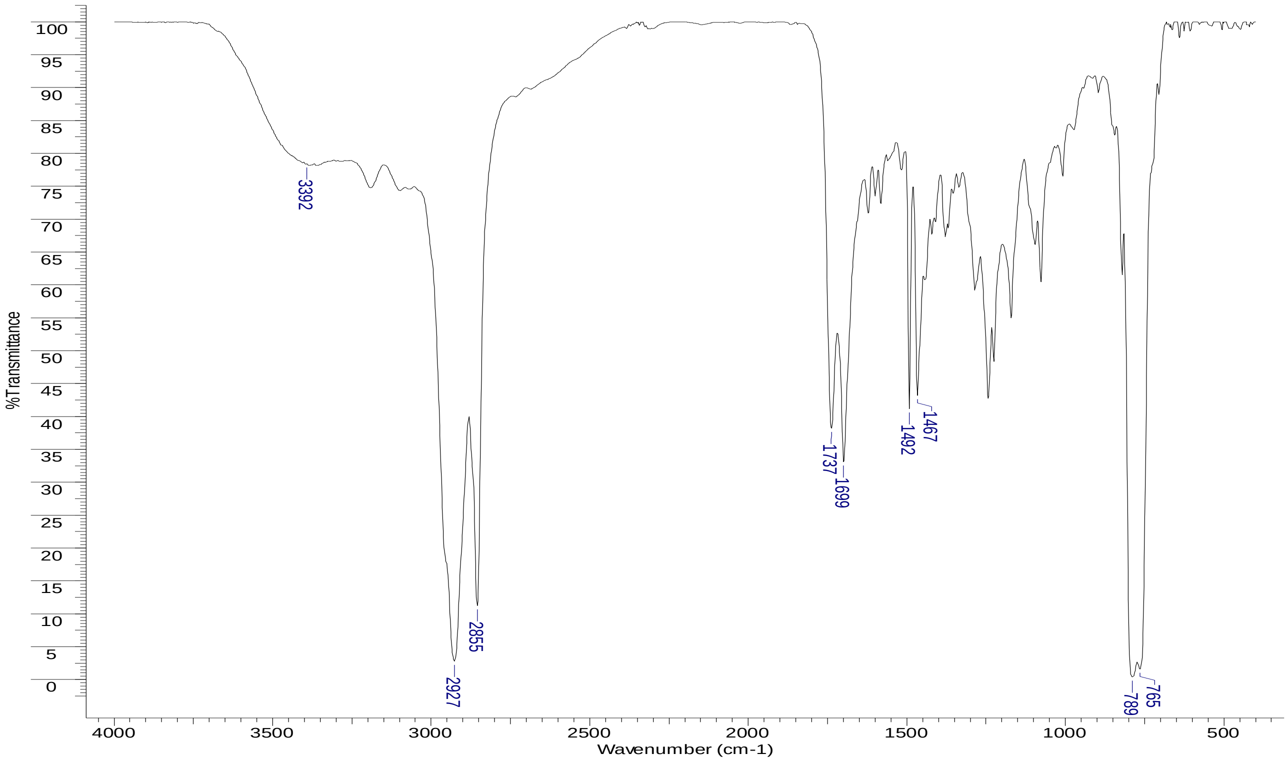

Figure S7. FT-IR spectrum of sample containing verbenone hydrazone **3** and lipids isolated from SC.

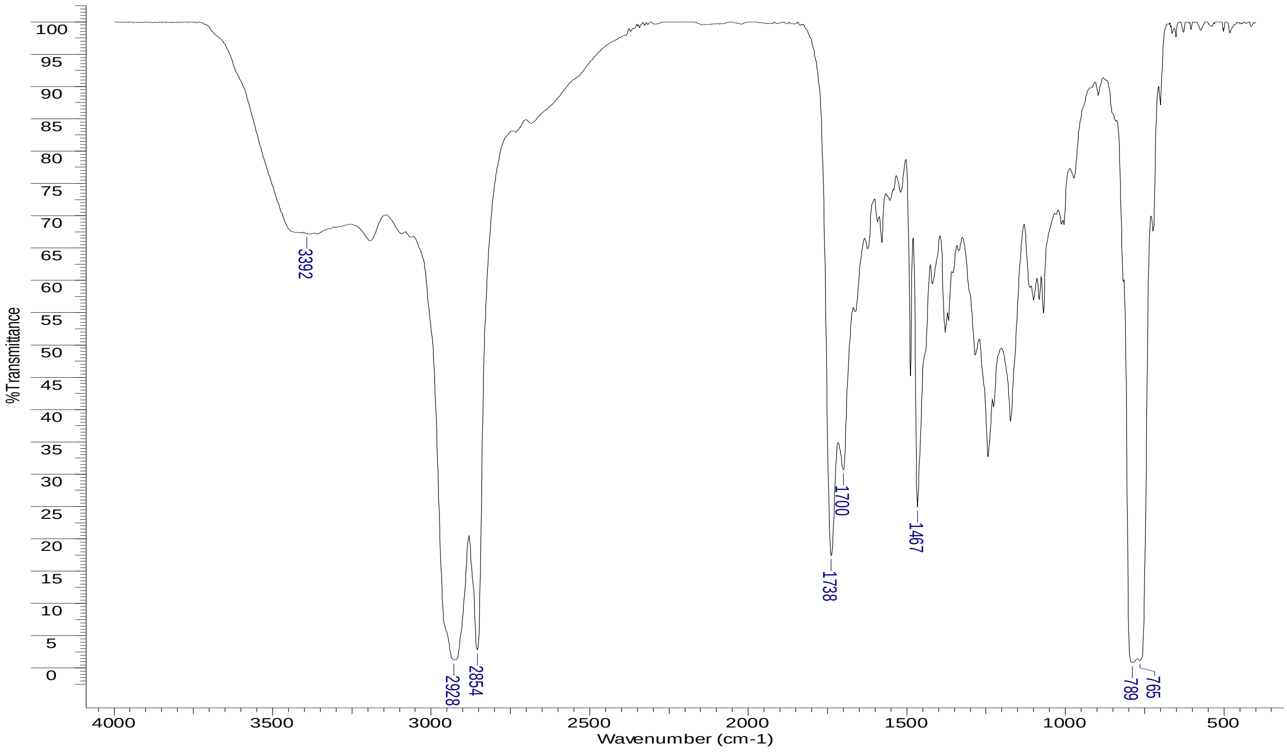

Figure S8. FT-IR spectrum of sample containing verbenone hydrazone **4** and lipids isolated from SC.

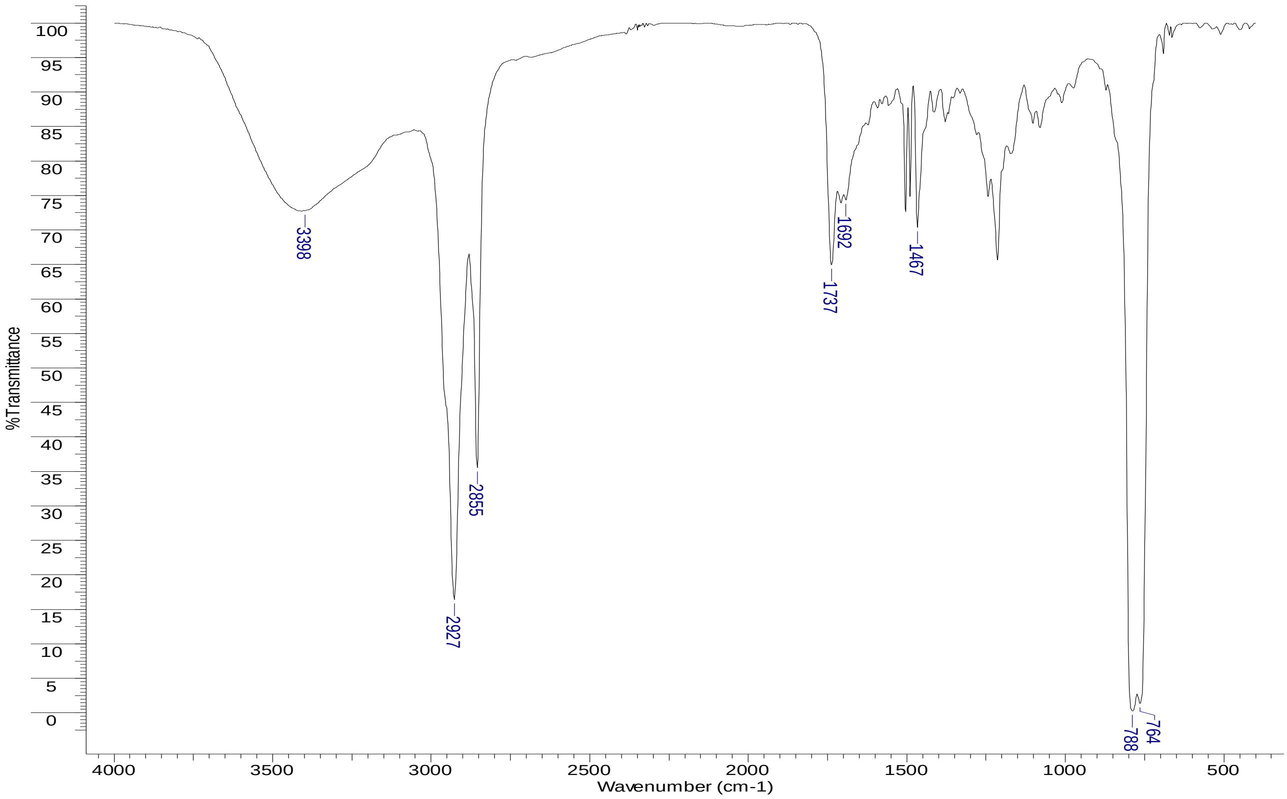

Figure S9. FT-IR spectrum of sample containing verbenone hydrazone **5** and lipids isolated from SC.

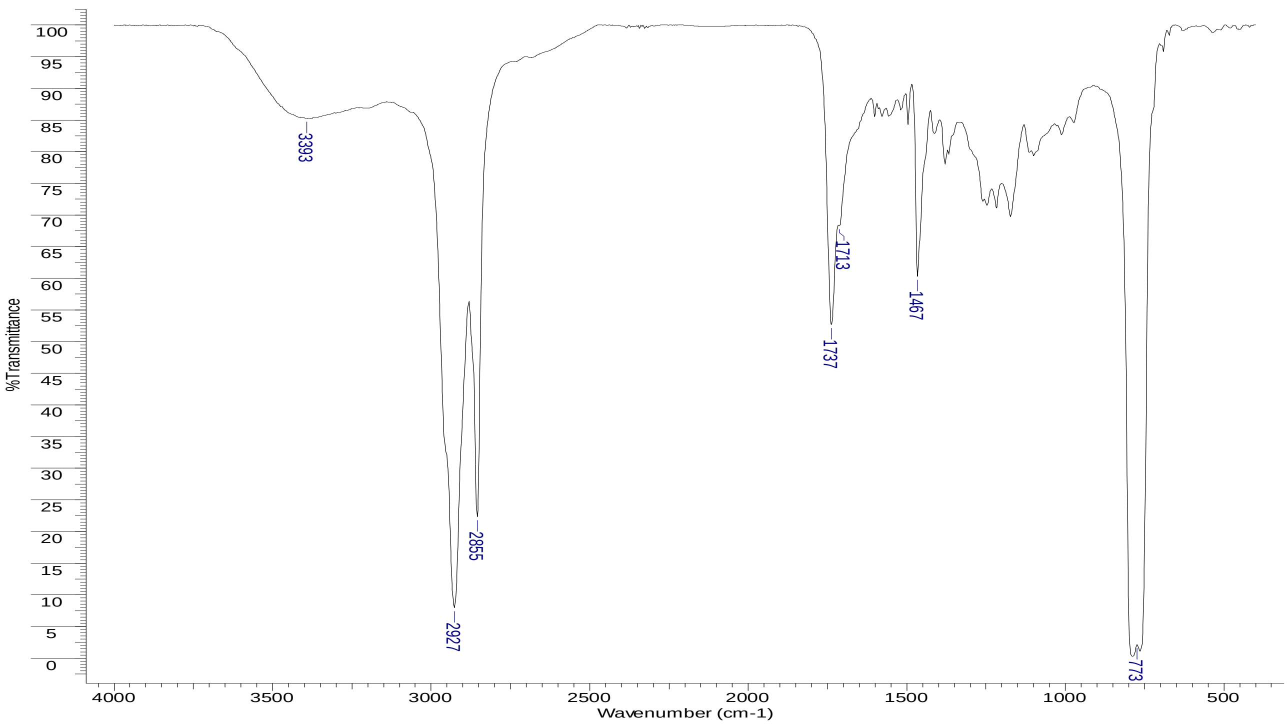

Figure S10. FT-IR spectrum of sample containing menthone hydrazone **6** and lipids isolated from SC.

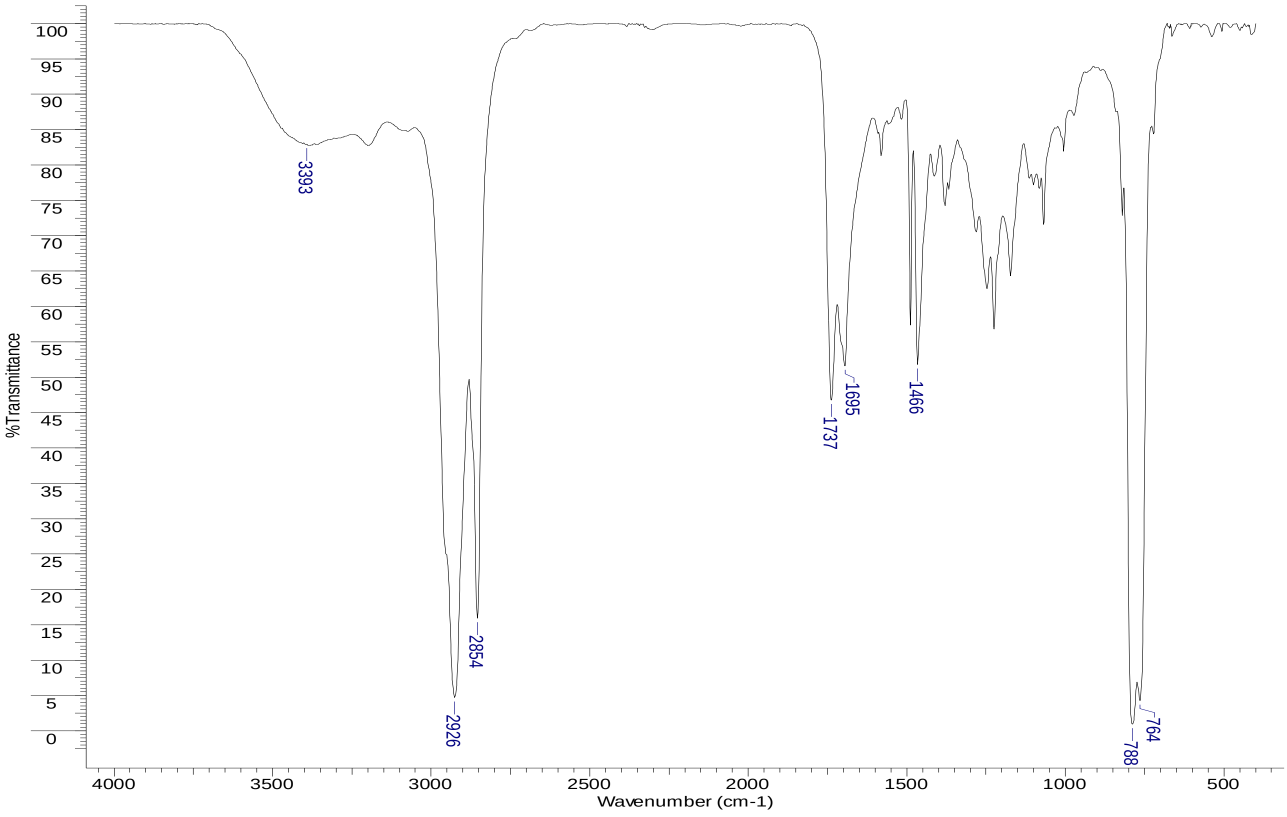

Figure S11. FT-IR spectrum of sample containing menthone hydrazone **7** and lipids isolated from SC.

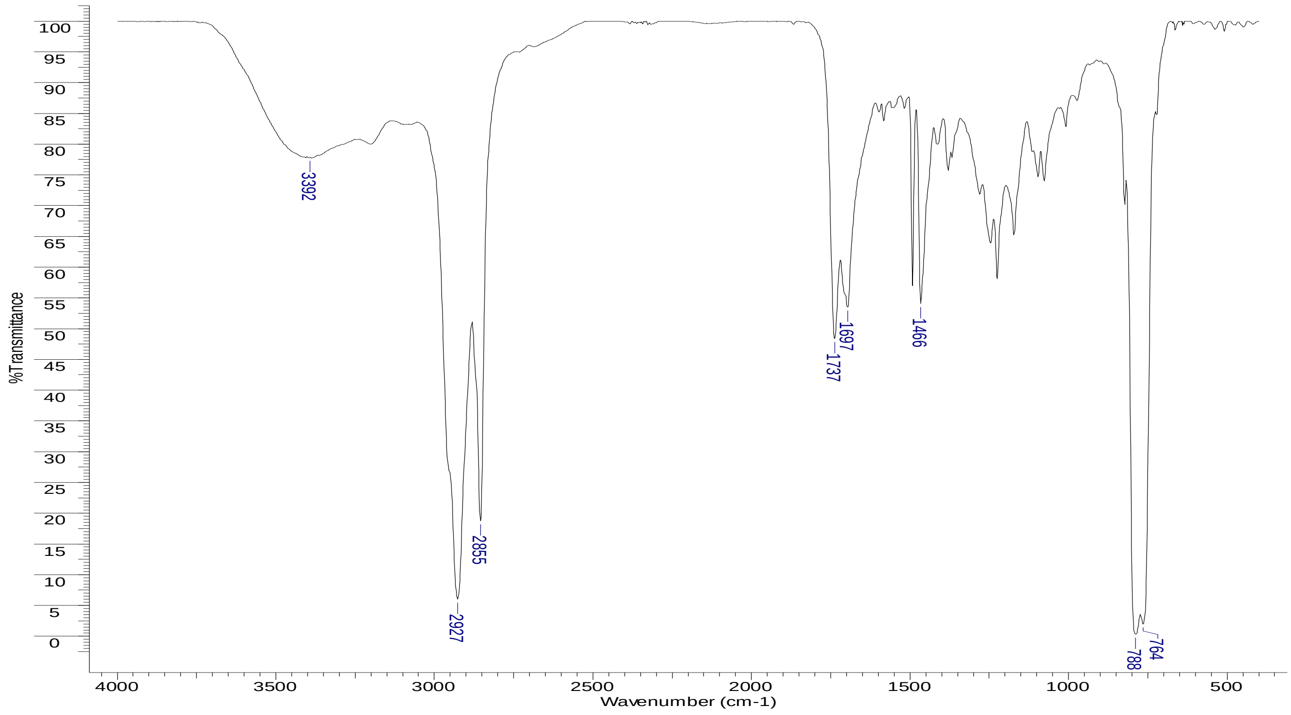

Figure S12. FT-IR spectrum of sample containing menthone hydrazone **8** and lipids isolated from SC.

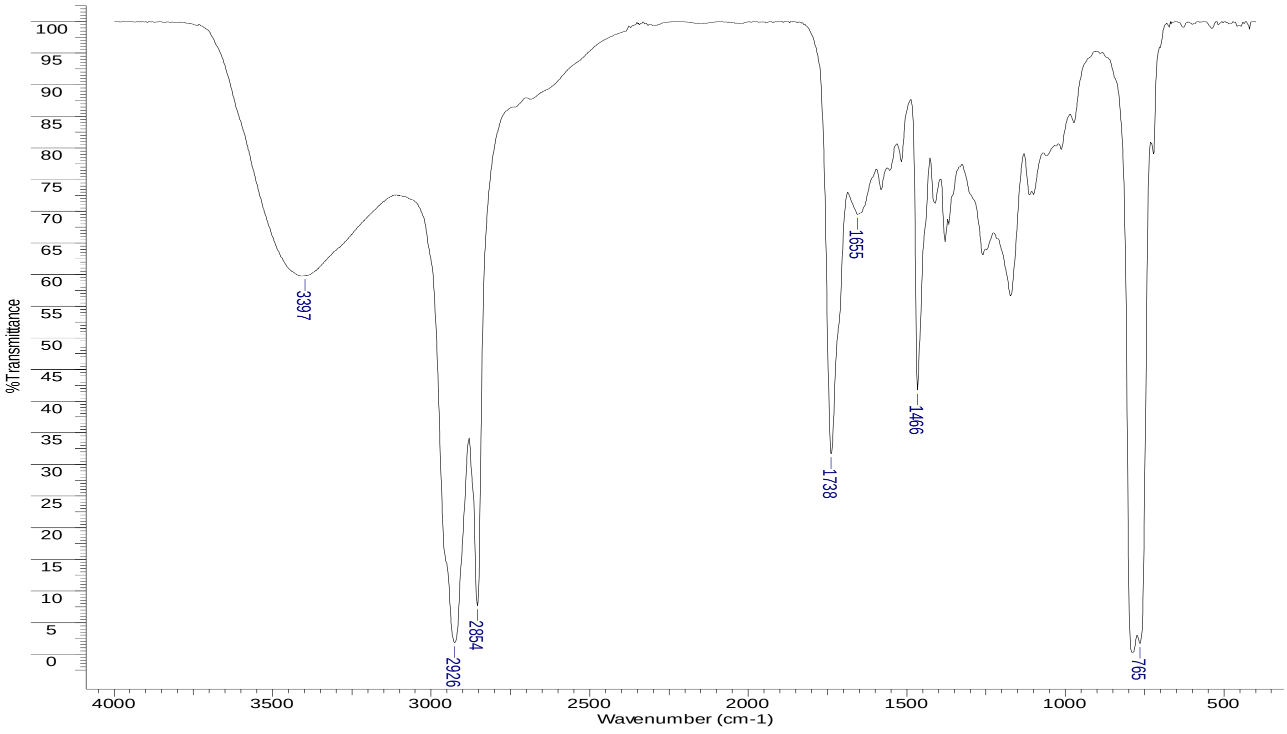

Figure S13. FT-IR spectrum of sample containing menthone hydrazone **9** and lipids isolated from SC.

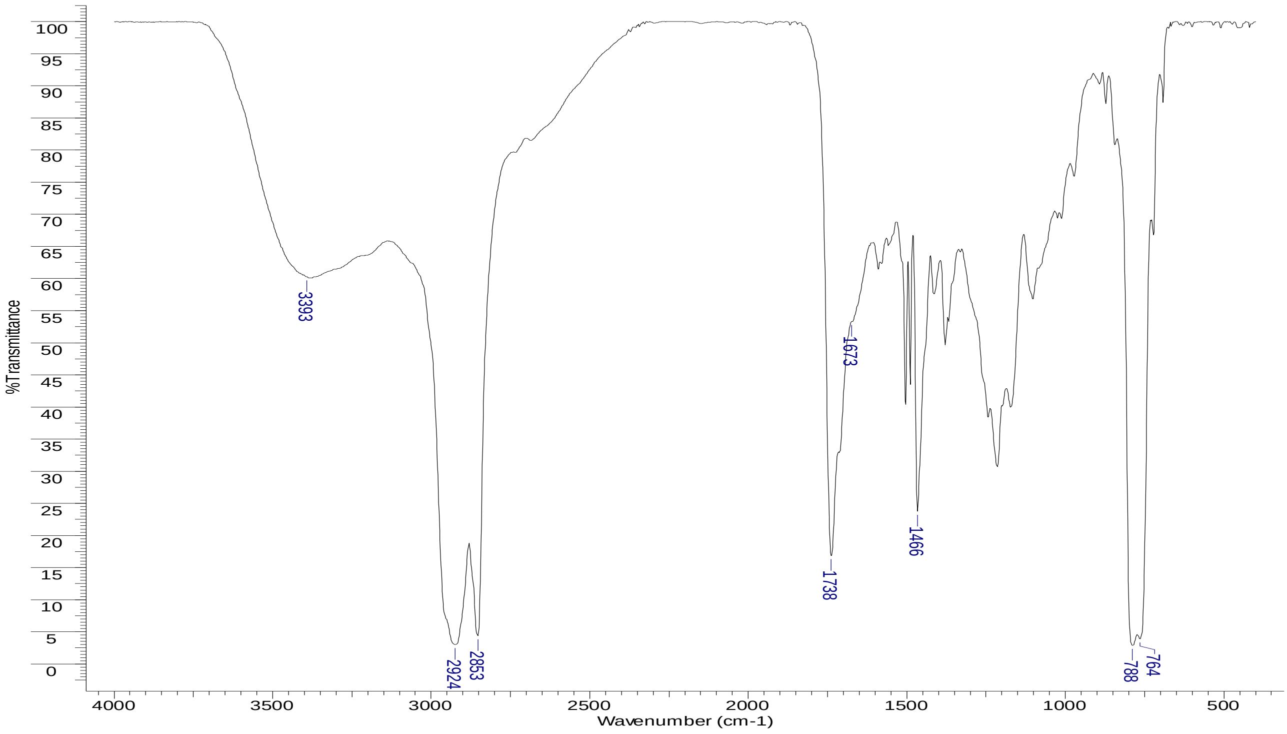

Figure S14. FT-IR spectrum of sample containing menthone hydrazone **10** and lipids isolated from SC.

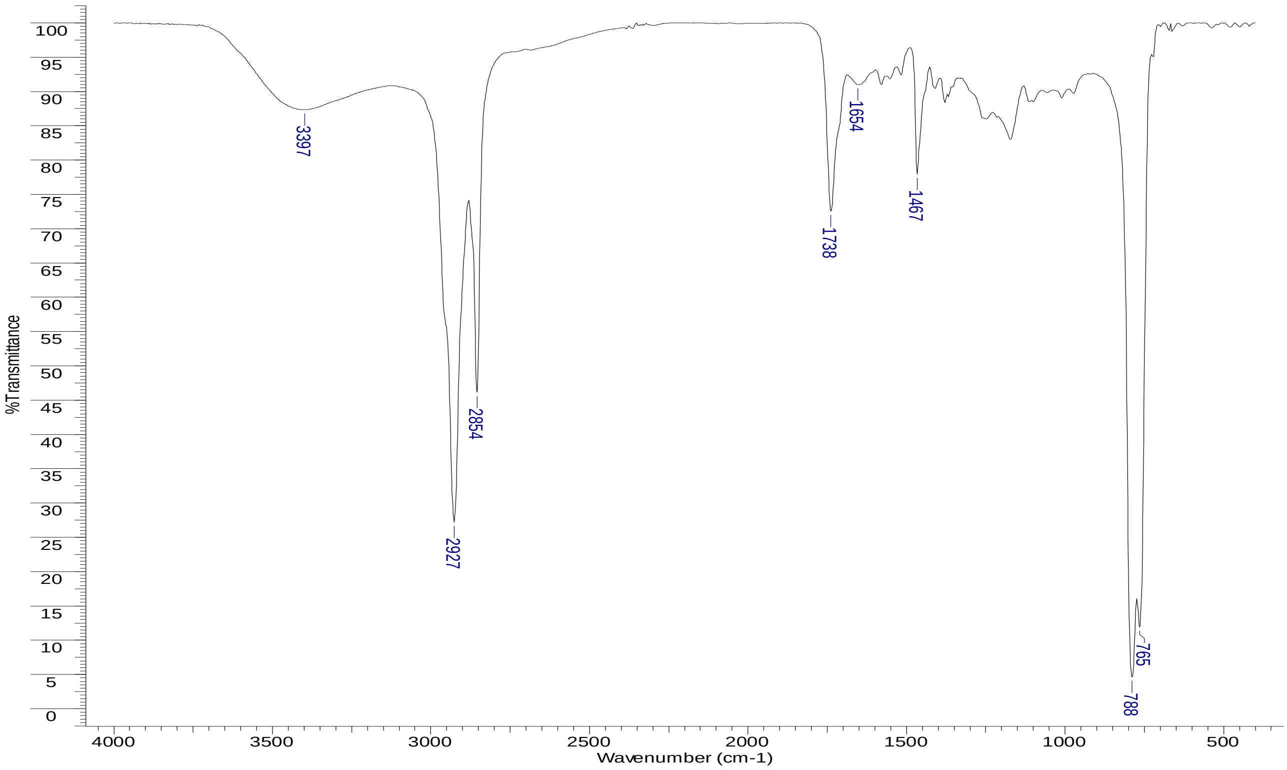

Figure S15. FT-IR spectrum of sample containing carvone hydrazone **11** and lipids isolated from SC.

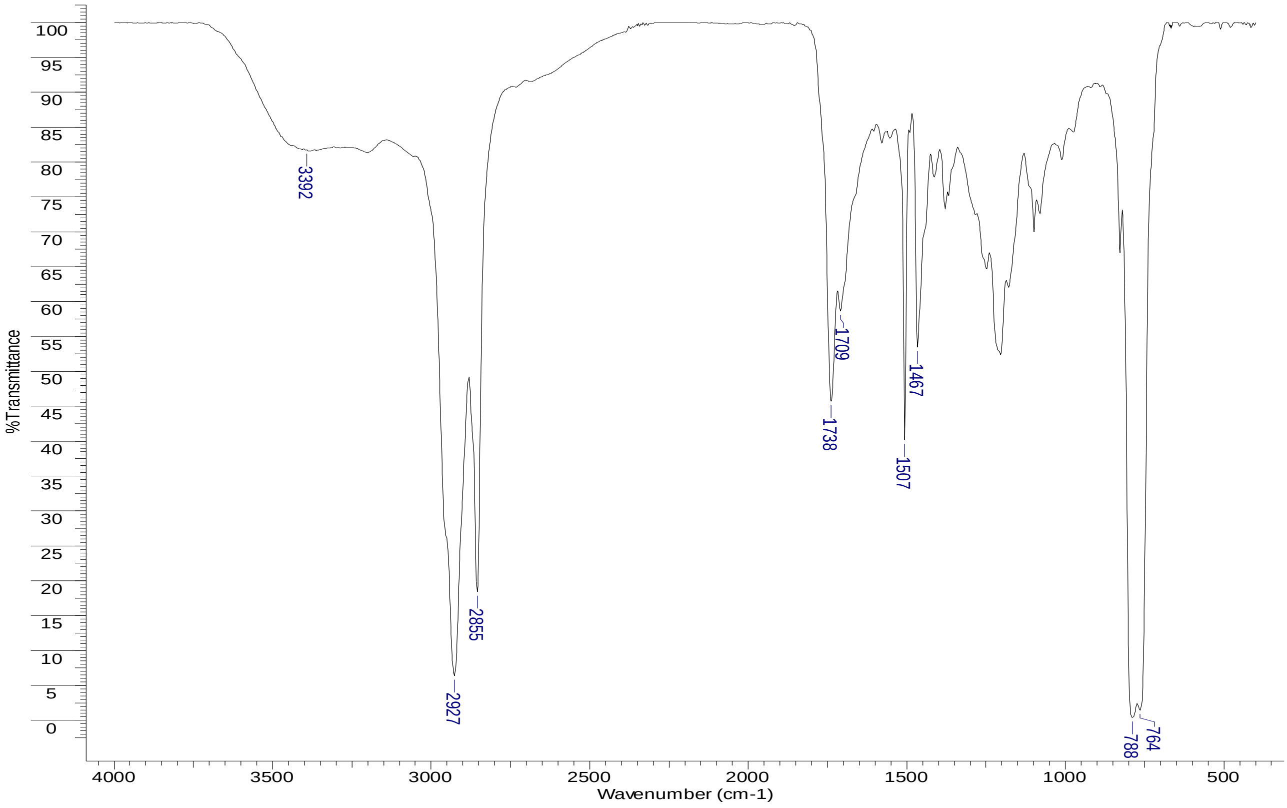

Figure S16. FT-IR spectrum of sample containing carvone hydrazone **12** and lipids isolated from SC.

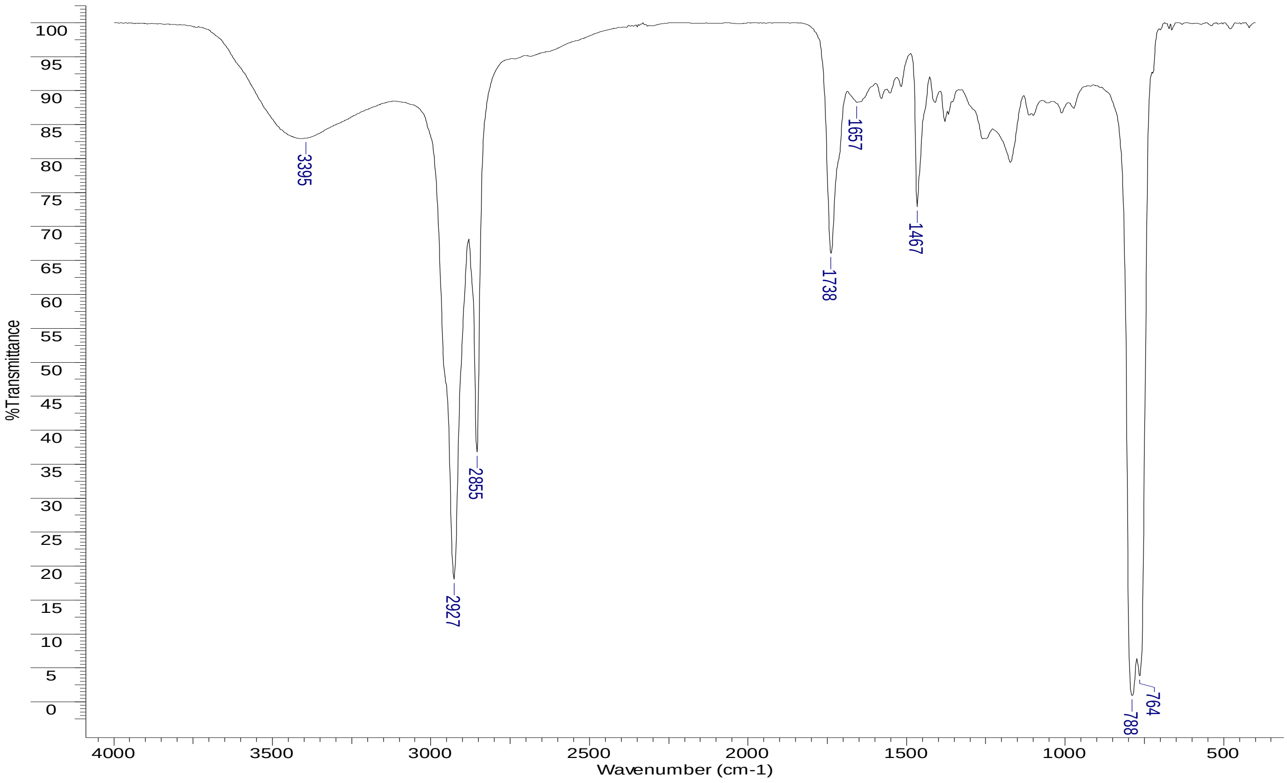

Figure S17. FT-IR spectrum of sample containing carvone hydrazone **13** and lipids isolated from SC.

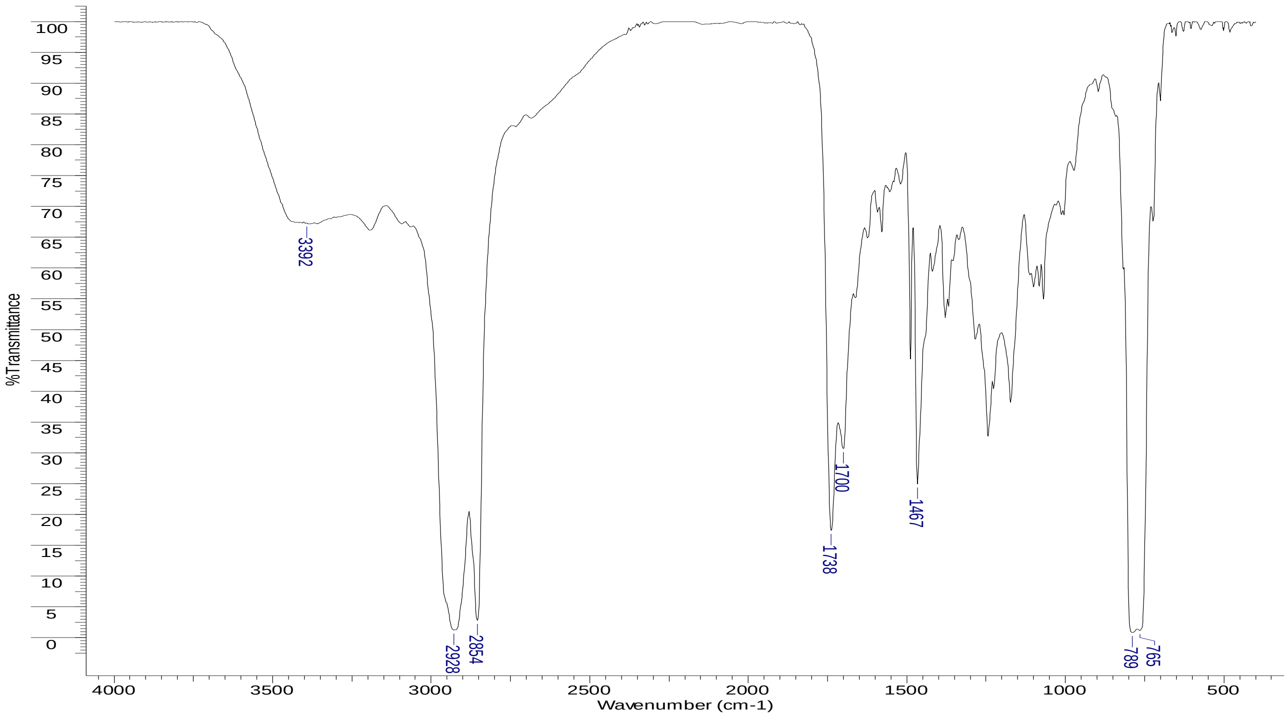

Figure S18. FT-IR spectrum of sample containing carvone hydrazone **14** and lipids isolated from SC.

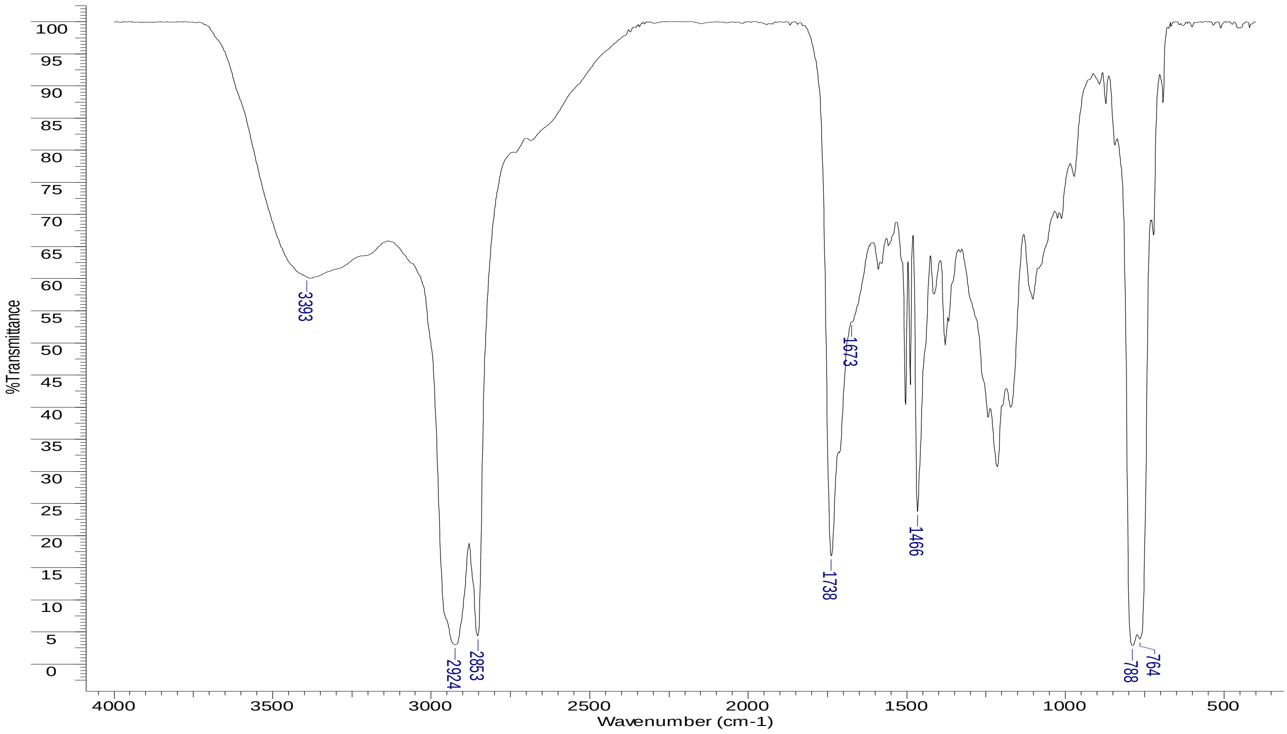

Figure S19. FT-IR spectrum of sample containing carvone hydrazone **15** and lipids isolated from SC.
